# Changes in Postpartum Opioid Prescribing After Implementation of State Opioid Prescribing Limits

**DOI:** 10.1001/jamahealthforum.2024.4216

**Published:** 2024-11-01

**Authors:** Carrie E. Fry, Alvin D. Jeffery, Manuel Horta, Yixuan Li, Sarah S. Osmundson, Julia Phillippi, Lori Schirle, Jake R. Morgan, Ashley A. Leech

**Affiliations:** Department of Health Policy, Vanderbilt University School of Medicine, Nashville, Tennessee; Vanderbilt University School of Nursing, Nashville, Tennessee; Department of Biomedical Informatics, Vanderbilt University School of Medicine, Nashville, Tennessee; Department of Health Policy, Vanderbilt University School of Medicine, Nashville, Tennessee; Department of Health Policy, Vanderbilt University School of Medicine, Nashville, Tennessee; Department of Obstetrics and Gynecology, Vanderbilt University School of Medicine, Nashville, Tennessee; Vanderbilt University School of Nursing, Nashville, Tennessee; Vanderbilt University School of Nursing, Nashville, Tennessee; Department of Health Policy, Law, and Management, Boston University School of Public Health, Boston, Massachusetts; Department of Health Policy, Vanderbilt University School of Medicine, Nashville, Tennessee

## Abstract

**IMPORTANCE:**

In response to the growing opioid crisis, states implemented opioid prescribing limits to reduce exposure to opioid analgesics. Research in other clinical contexts has found that these limits are relatively ineffective at changing opioid analgesic prescribing.

**OBJECTIVE:**

To examine the association of state-level opioid prescribing limits with opioid prescribing within the 30-day postpartum period, as disaggregated by type of delivery (vaginal vs cesarean) and opioid naivete.

**DESIGN, SETTING, AND PARTICIPANTS:**

This retrospective, observational cohort study used commercial claims data from January 1, 2014, to December 31, 2021, from 49 US states and a difference-in-differences staggered adoption estimator to examine changes in postpartum opioid prescribing among all deliveries to enrollees between the ages of 18 and 44 years in the US.

**EXPOSURES:**

The implementation of a state opioid prescribing limit between 2017 and 2019.

**MAIN OUTCOMES AND MEASUREMENTS:**

The primary outcomes for this analysis were the number of prescriptions for opioid analgesics, proportion of prescriptions with a supply greater than 7 days, and milligrams of morphine equivalent (MMEs) per delivery between 3 days before and 30 days after delivery.

**RESULTS:**

A total of 1 572 338 deliveries (enrollee mean [SD] age, 30.20 [1.59] years) were identified between 2014 and 2021, with 32.3% coded as cesarean deliveries. A total of 98.4% of these were to opioid-naive patients. The mean MMEs per delivery was 310.79, with higher rates in earlier years, states that had an opioid prescribing limit, and cesarean deliveries. In a covariate-adjusted difference-in-differences regression analysis, opioid prescribing limits were associated with a decrease of 148.70 MMEs per delivery (95% CI, −657.97 to 360.57) compared with states without such limits. However, these changes were not statistically significant. The pattern of results was similar among other opioid-prescribing outcomes and types of deliveries.

**CONCLUSIONS AND RELEVANCE:**

The results of this cohort study suggest that opioid prescribing limits are not associated with changes in postpartum opioid prescribing regardless of delivery type or opioid naivete, which is consistent with research findings on these limits in other conditions or settings. Future research could explore what kinds of prevention mechanisms reduce the risk of opioid prescribing during pregnancy and postpartum.

## Introduction

During the past 3 decades, the opioid crisis has devastated communities in the US. As a result, opioid use (in combination with mental health conditions) has substantially contributed to the rise in pregnancy-associated morbidity and mortality in the US, leading to maternal outcomes worse than those of other high-income nations.^1^ Additionally, pregnancy and postpartum opioid use substantially affects the next generation, as it is associated with an increased risk of in utero exposure to opioids, unused opioids in the home, and parental substance use.

One potential way to curb the opioid crisis is to restrict access to prescription opioid analgesics, thereby preventing exposure to opioids for patients and their families and reducing the risk opioid-related harms.^2^ Opioid prescribing limits, which limit the supply or dosage quantities of opioid analgesics to opioid-naive patients or patients after certain health care interactions, are one approach to restricting access to these medications.

Previous research on postpartum opioid prescribing interventions included single-site or single-state evaluations of limits, guidelines, or quality improvement practices and generally found declines in postpartum opioid prescribing.^3–7^ Multistate evaluations of opioid prescribing limits using national-level claims databases have occurred primarily in other clinical conditions and found no association between the policy adoption and opioid prescribing generally or specifically for nonpregnant populations.^8–13^

Postpartum opioid prescribing is common following vaginal and cesarean deliveries^14–16^ and has been associated with persistent use of opioid analgesics, as well as increased risk of opioid use disorder diagnosis, opioid-related overdose, or opioid-related death.^14–17^ These risks increase with higher dosages of the initial prescription and the number of opioid prescriptions filled.^15,17^ Thus, obstetricians may be more likely to adjust their prescribing habits in response to opioid prescribing limits, especially for vaginal deliveries, for which opioids are not commonly indicated. Yet, the association of prescribing limits with postpartum prescribing has not been well studied, despite the pressing need to reduce maternal morbidity and mortality and the overall effects of opioids on families. In this study, we used commercial health insurance claims data and a difference-in-differences (DID) study design to examine the association between the implementation of opioid prescribing limits and postpartum opioid prescribing.

## Methods

### Data Source and Sample

We used a commercial claims database (MarketScan; Merativ) that includes enrollment data and medical/pharmacy claims for a population of commercially insured people in the US younger than 65 years in all 50 states and Washington, DC. This study was deemed exempt by the Vanderbilt University institutional review board and followed all Strengthening the Reporting of Observational Studies in Epidemiology (STROBE) analysis and reporting requirements for observational studies.

Using a previously validated algorithm and diagnosis and procedure codes, we identified all deliveries to enrollees between age 18 and 54 years in the commercial claims database between 2014 and 2021 (eAppendix and eTable 1 in Supplement 1).^18^ Data from 2013 were used as a lookback period for deliveries that occurred during the first quarter of 2014. We further identified deliveries by type (vaginal vs cesarean; eTable 2 in Supplement 1). For the relatively small number of deliveries (approximately 5%) that did not have a diagnosis or procedure code specifying vaginal vs cesarean delivery, we assumed that the deliveries were vaginal in the main analyses, as there are billing incentives for clinicians to code cesarean deliveries when they occur. Our results were robust to this decision. We also identified predelivery opioid status. Enrollees were considered opioid naive if they did not have an opioid prescription during the 3 months before delivery; all others were considered nonnaive.

We required enrollees to be continuously enrolled in insurance coverage 3 months before and 1 month after the date of delivery to measure previous opioid exposure and comorbid conditions. Multiple deliveries from the same person were included if they met the inclusion criteria for both pregnancies. We excluded deliveries that could not be attributed to a state by the employee geographic location provided in the commercial claims database.

### Exposure and Outcomes

In line with previous studies, we defined opioid prescribing limits as those that restrict the quantity or dosage of opioid analgesics that can be prescribed to a patient at one time.^8–11,19^ We included all laws that met this definition and were implemented between July 1, 2016, and January 1, 2019 (eTable 3 in Supplement 1). Using these criteria, 36 states implemented an opioid prescribing limit (ie, the treatment or exposure group) that applied to adults. The 12 states (plus Washington, DC) that had not implemented an opioid prescribing limit by January 1, 2019, and Nebraska (whose prescribing limit only applied to minors) composed the comparison group. The limit in Illinois was implemented in 2012, and Hawaii’s limit only applied to benzodiazepine and opioid coprescribing; both states were excluded from this analysis.

We identified all opioid analgesic prescriptions indicated for treating pain that were filled during the 30 days after delivery (eFigure 1 and eTable 4 in Supplement 1). The outcomes were the (1) total number of opioid prescriptions per delivery, (2) proportion of prescriptions with a supply greater than 7 days, (3) number of milligrams of morphine equivalent (MMEs) prescribed per delivery, and (4) number of MMEs per prescription. As most of these limits restricted each prescription to a 7-day supply of opioids, clinicians could compensate for the introduction of these limits by writing more prescriptions for shorter durations or by substituting more potent formulations of opioid pain relievers. The US Centers for Disease Control and Prevention’s Opioid and Oral MME Conversion were use to standardize the dosages of each opioid prescription.^20^

We excluded monoproduct buprenorphine products (ie, Probuphine and Subutex) and buprenorphine-naloxone combination products, as these are commonly used to treat opioid use disorder rather than pain. We included any opioid analgesic prescriptions filled 3 days before and 30 days after the delivery date to account for scheduled deliveries for which opioids may be prescribed in advance.^9,21^

### Statistical Analysis

We conducted a staggered treatment timing DID analysis to compare changes in outcomes in states that implemented a limit with states that did not before and after the limit was implemented. A 2-way fixed-effects (TWFE) DID design assumes that the untreated states are a reasonable counterfactual for what would have happened in the treated states without a limit. Previous literature has suggested that using a TWFE DID with multiple adoption points may result in bias.^22^ To improve validity and decrease bias, we used the estimator proposed by Callaway & Sant’Anna,^23^ as it can account for staggered implementation and group-varying and time-varying treatment effects when there are more than 5 treated states per period. This estimator extends the strict parallel trends assumption to include covariates; conditional on covariates, the comparison group represents a reasonable counterfactual for the treated group absent treatment. We implemented the doubly robust estimator, which uses stabilized inverse propensity treatment weighting regression in addition to the outcome regression. In a doubly robust analysis, only 1 of these regressions must be correctly specified to produce unbiased estimates.

The Callaway & Sant’Anna estimator does not have an option to conduct nonlinear regression models, so we used linear regression models with standard errors clustered at the state level (the level of intervention). Additionally, staggered adoption estimators allow researchers the choice of comparison groups; we included not yet and never treated states (ie, the not yet treated option) as the comparison group in the main analyses. Not yet treated states were more like the states that adopted an opioid prescribing limit in terms of opioid burden and willingness to implement such a limit.

In DID, confounders are things that covary with the outcome or exposure over time and by group.^24^ Measurable confounders should be included as covariates in DID regression models to address the bias induced by them. In our study, we included a measure of substance use disorder prevalence in the population of interest, as increased rates of substance use disorder maybe associated with a state’s propensity to implement a prescribing limit and the amount of opioid pain relievers prescribed. Other covariates may be included to increase the precision of estimates. In our analysis, we include age in years at delivery, a pregnancy-related comorbidity score,^25^ and the prevalence of comorbid mental health conditions to help with the precision of our estimates (eTable 4 in Supplement 1).

We also conducted 2 analyses to test the mechanisms through which changes in opioid prescribing may be occurring. First, we stratified our analysis by type of delivery. Opioid prescriptions for vaginal deliveries may be more responsive to prescribing limits, as these medications are generally not indicated for a vaginal delivery.^26^ Additionally, we stratified our analyses by opioid-naive status as most limits only apply to opioid-naive patients. Thus, we would expect to see a clearer and stronger signal among deliveries to opioid-naive patients. Because some of the implementation dates had 5 or fewer treated units (2016 had 3, 2019 had 5) and the Callaway & Sant’Anna estimator may underperform in these situations, we also implemented 2 other staggered adoption estimators, a stacked regression DID estimator and the extended TWFE estimator proposed by Wooldridge,^27,28^ to examine the robustness to our choice of staggered adoption estimator. In the stacked regression DID, we included the 2 years before and after the limit was implement in each subexperiment. For comparison with Callaway & Sant’Anna, we chose a linear regression model in the extended TWFE and stacked DID regression estimators.

We considered statistical significance to be at the α = .05 level. Cohort construction was done in SAS, version 3.81 (SAS Institute), and statistical analyses were conducted in R (version 2023.09.1+494; R Foundation).

## Results

We identified 1 572 338 deliveries in the commercial claims database between 2014 and 2021; of those, 508 615 (32.3%) were cesarean deliveries and 1 063 723 (67.7%) were vaginal deliveries. Most deliveries (1 547 386 [98.4%]) were to opioid-naive patients. Before any state in our study implemented an opioid prescribing limit (2014–2015), states that would eventually implement a limit had nearly 3 times more deliveries than states without a limit (374 126 vs 124 823; Table 1). However, the proportion of vaginal and opioid-naive deliveries were similar between states that would and would not eventually adopt an opioid prescribing limit. Enrollees who delivered in states that implemented a limit were similar in age at delivery and comorbidities compared with enrollees who delivered in comparison states (Table 1) before 2016.

Overall, postpartum opioid prescribing was considerably higher after cesarean deliveries and for non–opioid-naive enrollees compared with vaginal deliveries and opioid-naive enrollees, respectively, regardless of the presence of an opioid prescribing limit (Table 1). In general, states that eventually implemented an opioid prescribing limit during our study prescribed more opioids (with respect to MMEs) per delivery and per prescription, although these differences were not statistically significant. The proportion of prescriptions with a day’s supply greater than 7 were similar in states regardless of opioid prescribing limit presence. During the entire study period, unadjusted MMEs per delivery and opioid prescription were higher in states with compared with states without a limit. MMEs per delivery and per prescription declined substantially between 2014 and 2021 among all states in the sample (Figure). States that adopted a prescribing limit experienced an uptick in MMEs per prescription and delivery in 2020 compared with states that did not.

### DID Estimates

In covariate-adjusted DID regression analysis, the rate of opioid prescribing within the first 30 days postpartum decreased among all deliveries by 148.70 MMEs per delivery (95% CI, −657.97 to 360.57) in states that adopted an opioid prescribing limit compared with states that had not yet adopted such a limit (Table 2). This decline was not statistically significant. States that implemented a prescribing limit in 2017 experienced the greatest decline in MMEs per delivery (−323.94; 95% CI, −1266.39 to 618.52), while states that implemented in 2018 experienced an increase in MMEs per delivery (70.35; 95% CI, −61.15 to 200.86). Neither difference was statistically significant. Similarly, the MMEs per prescription declined by 104.41 MMEs (95% CI, −608.65 to 399.84) overall, in 2017 (−232.62; 95% CI, 1296.90–930.06), and in 2019 (−33.04; 95% CI, −93.68 to 27.59); none of these changes were different from 0.

Prescribers may have adjusted their opioid prescribing along other margins to be within new limits while maintaining patient access to opioid pain relievers. To be within day-supply constraints (how many states’ limits are defined), prescribers may have written more prescriptions for a shorter duration per delivery. However, we found no statistically significant change in the number of prescriptions per delivery (−0.06; 95% CI, −0.15 to 0.04) or the proportion of prescriptions with a greater than 7-day supply (−0.001; 95% CI, −0.03 to 0.03) (Table 3). These outcomes met the conditional parallel trends assumption (Table 3; eFigure 2 in Supplement 1).

Changes in prescribing by delivery type may be obscured by the average treatment effects. Postpartum opioid prescribing was higher among cesarean deliveries in all states with potentially more room for decreases. Conversely, the adoption of a limit may have alerted prescribers to the lack of indication for most vaginal deliveries. Postpartum opioid prescribing among cesarean deliveries declined after the adoption of a prescribing limit (−242.27; 95% CI, −1174.04 to 689.51) in states that adopted a limit compared with those that did not (Table 3). Declines in limit states were smaller among vaginal deliveries and not statistically significant (−90.45; 95% CI, −441.57 to 260.67).

Additionally, most limits only applied to patients receiving new opioid prescriptions. So, we might expect to see larger and more precisely estimated changes among opioid-naive enrollees. When we stratified by opioid naivete, we estimated nonstatistically significant declines in MMEs per delivery (−106.25; 95% CI, −595.83 to 383.32) and prescription (−124.31; 95% CI, −827.37 to 578.74) among opioid-naive patients in states that adopted a prescribing limit compared with states that did not. Caution is warranted in interpreting both results for opioid-naive patients, as neither outcome meets the parallel trends assumption (Table 3).

### Robustness and Sensitivity Analyses

Unadjusted regression estimates reflected the same pattern of results as the covariate-adjusted estimates; the unadjusted regression estimates were roughly 20% smaller than the covariate-adjusted estimates (eTable 6 in Supplement 1). Finally, our unadjusted estimates using the extended TWFE and stacked DID estimators (−75.5 and −165.79 MMEs per delivery, respectively; eTable 7 in Supplement 1) were very similar to that produced by Callaway & Sant’Anna, providing evidence that our results are not sensitive to the estimator chosen.

## Discussion

In this cohort study, we examined the association between the adoption of an opioid prescribing limit and postpartum opioid prescribing. Overall, we did not find any statistically significant changes in several measures of postpartum opioid prescribing in states that implemented an opioid prescribing limit compared with states that had not implemented. However, the confidence intervals from many of these estimates cannot eliminate the possibility of clinically meaningful changes in MMEs per delivery and per prescription. Our confidence intervals suggested changes in the number of oxycodone tablets per delivery to vary by as much as 132 fewer to 72 more tablets.

The overall estimates reflected the broader pattern of results seen in evaluations of opioid prescribing limits using DID in other patient populations. Studies using populations other than postpartum individuals operationalized outcomes differently (eg, proportion of people with a prescription, number of days covered) but found the same pattern of results. Among children and adolescents, an opioid prescribing limit was associated with a 0.08–percentage point (95% CI, −0.3 to 0.4) increase in the number of opioid prescriptions filled.^11^ Among patients with chronic pain, an implemented opioid prescribing limit was associated with insignificant and small declines in the proportion of the patient population prescribed an opioid (0.005; 95% CI, 0.01–0.004) and the average number of opioid prescriptions (0.02; 95% CI, −0.12 to 0.08).^10^ This study also found a decrease of 2.2 MMEs per day (95% CI, −5.13 to 0.78), which may be a clinically significant decline, as 2.2 MMEs represent roughly half of an oxycodone pill per day. However, this latter study required patients to be continuously enrolled in administrative claims data for 7 years, limiting generalizability. In a postsurgical population, opioid prescribing limits were associated with a decline in days’ supply of opioids but were not associated with changes in the number or daily dose of opioids.^9^ In another study using a commercially insured adult population, opioid prescribing limits were associated with declines (1.9 MMEs per day per patient per year; 95% CI, −4.1 to 0.4) in opioid prescribing.^8^ Differences between our results and the results of other literature may be explained by the nature of these limits.

There has been a growing body of literature and consensus that opioid prescribing for most vaginal and some cesarean deliveries is not only unwarranted but also risky for postpartum people, resulting in harm to this population.^14–17^ Our study found consistent declines in opioid prescribing during the first 30 days postpartum for vaginal and cesarean deliveries in states that implemented limits, even though rates of opioid prescribing in vaginal deliveries were considerably lower than for cesarean deliveries before a limit was implemented. However, none of our estimates were statistically significant. Future research should explore whether these limits are more effective in other settings in which opioid prescribing is not clinically indicated but occurs frequently.

One impetus for opioid prescribing limits was a US Centers for Disease Control and Prevention recommendation as a way for states to curb their opioid crisis. However, this study and others^8–12^ examining multiple states and national-level claims databases have found no change in opioid prescribing after the adoption of a limit. Another study that examined specific settings in which opioid prescribing may be more discretionary and less urgent (eg, outpatient adult and pediatric offices) found considerable reductions in opioid prescribing among Medicaid beneficiaries.^29^ Perhaps it is these types of settings in which opioid limits are most effective.

### Limitations

As is true with all observational study designs, our study had limitations. First, we used commercial insurance claims rather than Medicaid claims or an all-payer claims database; 40% of all deliveries are paid for by Medicaid, and pregnant people receiving Medicaid are more likely to fill an opioid prescription postpartum than pregnant people with commercial insurance.^14,30,31^ Thus, these results may be an underestimate of the association of these limits with opioid prescribing in the population overall. This was echoed by a recent article that found reductions in opioid prescribing after a limit was implemented among Medicaid beneficiaries. Second, states implemented other policies around the same time to address the opioid crisis. To the extent that these policies were implemented in the same states at the same time as opioid prescribing limits, our analytic strategy cannot differentiate between these policies and an opioid prescribing limit. Third, we could not assess any pain-related outcomes that occurred as a result of these limits. Finally, our analyses relied on counterfactual assumptions that are not directly testable; the test used by Callaway & Sant’Anna estimator is indirect.

## Conclusions

The results of this cohort study suggest that existing opioid prescribing limits are not associated with decreases in opioid analgesic prescribing in short-term clinical settings in which patients may experience considerable pain, postpartum or otherwise. Yet, the opioid crisis in the US has continued to account for substantial severe maternal morbidity and mortality. Mental health and substance use disorders are one of the factors associated with increased maternal morbidity and mortality.^32^ While prevention measures like opioid prescribing limits are important in curbing the use of opioid analgesics, many of these measures have mixed or limited evidence on opioid-related outcomes. However, epidemiological research has found that no level of opioids are necessarily safe during the postpartum period, suggesting that prevention mechanisms are necessary to fully reduce the risk of harm.^15–17^ Additional research is needed to understand what those policy mechanisms may be. Finally, policy changes focused on harm reduction and treatment for individuals already affected by opioids will be equally important in reducing pregnancy-related morbidity and mortality rates. Such measures should include increasing access to evidence-based treatment like medications for opioid use disorder for pregnant and birthing people.^33^

## Supplementary Material

Supplement 1 eTable 1. Diagnosis and procedure codes used for the identification of a delivery
eTable 2. Identification of vaginal vs. cesarean births
eTable 3. Status of state opioid prescribing limits used in analysis
eAppendix. Identification of opioid analgesics
eFigure 1. Logic followed for identification of opioid pain reliever NDCs in 2021
eTable 3. Generic opioid formulations included in the 2021 creation of the CDC Opioid Oral MME conversion table
eTable 4. Categories and scores for maternal comorbidity index
eTable 5. Diagnosis codes used for definition of mental health conditions and substance use disorder
eFigure 2. Event study plot for Callaway-Sant’Anna estimator using the primary outcome, MMEs per delivery
eTable 6. Unadjusted Callaway-Sant’Anna DID estimates compared to fully adjusted for the outcome, MMEs per delivery
eTable 7. Average treatment effects using alternative DID estimators eReferences.

Supplement 2 Data sharing statement

## Figures and Tables

**Figure.**
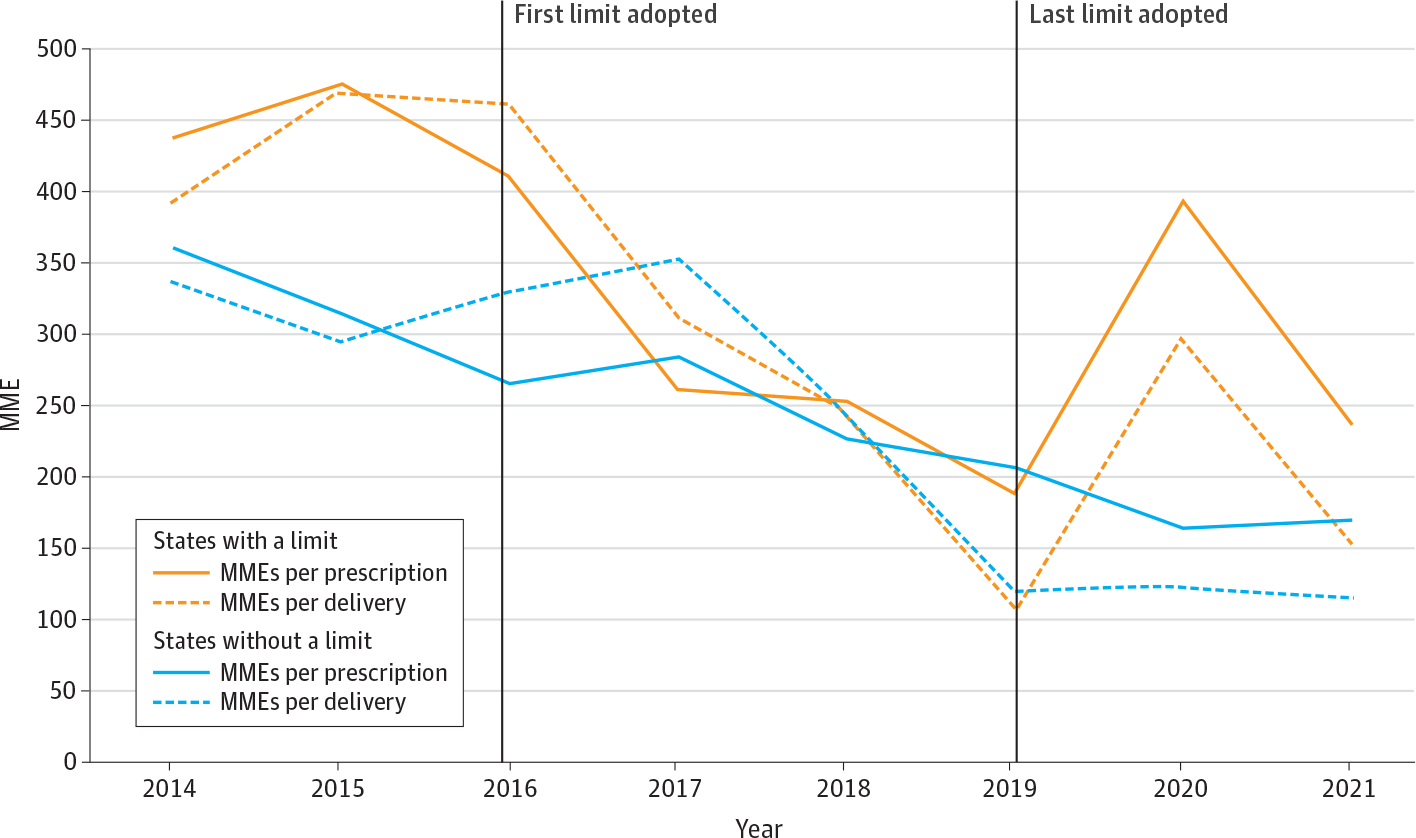
Unadjusted Opioid Prescribing During the 30 Days After Delivery by State Opioid Prescribing Limit From 2014 to 2021 Commercial claims database claims data from 2014 to 2021. Definitions and diagnosis/procedure codes for deliveries and type of delivery are available in the eAppendix in Supplement 1. Except when otherwise noted, values are means, and standard deviations are in parentheses. *t* Tests for a difference in means compared across rows. MME indicates milligrams of morphine equivalent.

**Table 1.** Characteristics by Opioid Prescribing Limit Status From 2014 to 2015

Characteristic	Mean (SD)	
	States with a limit	States without a limit
Delivery characteristics, No. (%)		
Total No. of deliveries	416 729	124 823
Vaginal deliveries	276 814 (66.43)	85 962 (68.87)
Cesarean deliveries	139 915 (33.57)	38 861 (31.13)
Opioid-naive deliveries	409 332 (98.22)	122 603 (98.22)
Non-opioid-naive deliveries	7397 (1.78)	2220 (1.78)
Enrollee characteristics		
Age at delivery, y	29.87 (1.15)	29.77 (1.35)
Mental health condition, %	7.72 (2.09)	7.61 (2.02)
Substance use disorder, %	1.68 (1.12)	1.21 (1.20)
Mean maternal comorbidity score	0.49 (0.06)	0.47 (0.10)
Postpartum opioid prescribing among all deliveries		
No. of prescriptions per delivery, all	0.95 (0.28)	0.91 (0.32)
MMEs per delivery, all	431.18 (532.86)	315.82 (301.81)
MMEs per prescription, all	455.82 (582.04)	388.29 (288.36)
Proportion of prescriptions with >7-d supply	0.18 (0.05)	0.17 (0.04)
Postpartum opioid prescribing by type of delivery		
MMEs per delivery, vaginal	271.16 (241.03)	231.18 (251.50)
MMEs per delivery, cesarean	799.25 (1326.26)	540.49 (511.00)
MMEs per delivery, opioid-naive enrollees	300.32 (384.84)	230.78 (230.45)
MMEs per delivery, non-opioid-naive enrollees	7324.95 (10 577.80)	4479.72 (4090.79)

Abbreviation: MME, milligrams of morphine equivalent.

**Table 2.** Changes in Postpartum Opioid Prescribing After the Implementation of an Opioid Prescribing Limit From 2014 to 2021

Characteristic	Estimate (95%CI)^a^	*P* value for nonparallel trends
No. of prescriptions per delivery		
ATT	−0.06 (−0.16 to 0.04)	
2017	−0.12 (−0.34 to 0.11)	.004
2018	0.03 (−0.09 to 0.14)	
2019	−0.05 (−0.20 to 0.10)	
MMEs per delivery		
ATT	−148.70 (−665.25 to 367.85)	
2017	−323.94 (−1356.35 to 708.48)	.17
2018	70.35 (−56.60 to 197.31)	
2019	−31.92 (−101.98 to 38.14)	
MMEs per prescription		
ATT	−104.41 (−679.39 to 470.57)	
2017	−232.62 (−1155.48 to 690.25)	.26
2018	59.92 (−41.05 to 160.90)	
2019	−33.04 (−93.17 to 27.09)	
Proportion of prescriptions >7 d supply		
ATT	0.001 (−0.30 to 0.04)	
2017	−0.005 (−0.07 to 0.06)	.37
2018	0.03 (−0.02 to 0.08)	
2019	−0.06 (−0.15 to 0.03)	

Abbreviations: ATT, overall average treatment effect; MME, milligrams of morphine equivalent.

a Values represent the covariate-adjusted difference-in-differences estimates using the Callaway-Sant’Anna doubly robust regression. Not yet and never treated states were included in the comparison group. Standard errors were clustered at the state level. Covariates included the mean age at delivery, the proportion of enrollees with a mental health condition, the proportion of enrollees with a substance use disorder, and the mean maternal comorbidity score. We identified opioid pain relievers by national drug codes. Calculations of MMEs for opioid pain relievers were calculated using the US Centers for Disease Control and Prevention 2020 conversion table by active ingredient and day supply. Estimates for years represent the group-time estimates(ie, the ATT estimate for states that implemented in the given year).

**Table 3.** Changes in Milligrams of Morphine Equivalent (MMEs) per Delivery and Prescription After the Implementation of Opioid Prescribing Limits by Type of Delivery and Opioid Naivete Status From 2014 to 2021

Characteristic	Estimate (95% CI)^a^	*P* value for nonparallel trends
Opioid-naive deliveries		
MMEs per delivery	−130.92 (−592.18 to 330.33)	.05
MMEs per prescription	−108.19 (−577.26 to 360.88)	.004
MMEs per vaginal delivery	−90.45 (−423.12 to 242.23)	.53
MMEs per cesarean delivery	−242.26 (−1176 to 692.34)	.63

a Values represent the covariate-adjusted difference-in-differences estimates (average treatment effects on the treated) from the Callaway & Sant’Anna staggered treatment timing estimator. Not yet and never treated states were included in the comparison group. Standard errors were clustered at the state level. Covariates included the mean age at delivery, proportion of enrollees with a mental health condition, proportion of enrollees with a substance use disorder, and mean maternal comorbidity score. We identified opioid pain relievers by national drug codes. Calculations of MMEs for opioid pain relievers are calculated using the US Centers for Disease Control and Prevention 2020 conversion table by active ingredient and day supply. Deliveries were identified as vaginal or cesarean using procedure codes during the 7 days before or after the delivery. Deliveries without identifying procedure codes were assumed to be vaginal due to the financial incentive to code cesarean deliveries. Opioid naivete was defined as not having a prescription for an opioid pain reliever during the 90 days before delivery.

## Key Points

### Question

How does postpartum opioid prescribing change after the adoption of an opioid prescribing limit?

### Findings

In this cohort study of 1 572 338 deliveries, the adoption of an opioid prescribing limit did not change several measures of postpartum opioid prescribing, regardless of delivery type or naivete status.

### Meaning

The study results suggest that opioid prescribing limits may not be the most effective way to reduce opioid prescribing to postpartum patients.

## References

[1] Gunja MZ, Gumas ED, Williams RD II. The U.S. maternal mortality crisis continues to worsen: An internationalcomparison. Accessed January 28, 2024. https://www.commonwealthfund.org/blog/2022/us-maternal-mortality-crisis-continues-worsen-international-comparison

[2] Lee B, Zhao W, Yang KC, Ahn YY, Perry BL. Systematic evaluation of state policy interventions targeting the USopioid epidemic, 2007–2018. JAMA Netw Open. 2021;4(2):e2036687. 33576816 10.1001/jamanetworkopen.2020.36687 PMC7881356

[3] Holland E, Bateman BT, Cole N, Evaluation of a quality improvement intervention that eliminated routineuse of opioids after cesarean delivery. Obstet Gynecol. 2019;133(1):91–97. 30531571 10.1097/AOG.0000000000003010

[4] Potnuru PP, Patel SD, Birnbach DJ, Epstein RH, Dudaryk R. Effects of state law limiting postoperative opioid prescription in patients after cesarean delivery. Anesth Analg. 2021;132(3):752–760. 32639388 10.1213/ANE.0000000000004993

[5] Prabhu M, McQuaid-Hanson E, Hopp S, A shared decision-making intervention to guide opioid prescribing after cesarean delivery. Obstet Gynecol. 2017;130(1):42–46. 28594762 10.1097/AOG.0000000000002094 PMC5482786

[6] Kim DD, Chiang E, Volio A, Reducing inpatient opioid consumption after caesarean delivery: effects of an opioid stewardship programme and racial impact in a community hospital. BMJ Open Qual. 2024;13(2):e002265. 10.1136/bmjoq-2023-002265 PMC11086205 38684344

[7] Phinn K, Liu S, Patanwala AE, Penm J. Effectiveness of organizational interventions on appropriate opioid prescribing for noncancer pain upon hospital discharge: A systematic review. Br J Clin Pharmacol. 2023;89(3):982–1002. 36495313 10.1111/bcp.15633

[8] Tormohlen KN, McCourt AD, Schmid I, State prescribing cap laws’ association with opioid analgesic prescribing and opioid overdose. Drug Alcohol Depend. 2022;240:109626. 36115221 10.1016/j.drugalcdep.2022.109626 PMC9893520

[9] Schmid I, Stuart EA, McCourt AD, Effects of state opioid prescribing cap laws on opioid prescribing after surgery. Health Serv Res. 2022;57(5):1154–1164. 35801988 10.1111/1475-6773.14023 PMC9441291

[10] McCourt AD, Tormohlen KN, Schmid I, Effects of opioid prescribing cap laws on opioid and other pain treatments among persons with chronic pain. J Gen Intern Med. 2023;38(4):929–937. 36138276 10.1007/s11606-022-07796-8 PMC10039157

[11] Stone EM, Tormohlen KN, McCourt AD, Association between state opioid prescribing cap laws and receipt-of opioid prescriptions among children and adolescents. JAMA Health Forum. 2022;3(8):e222461. 36003417 10.1001/jamahealthforum.2022.2461 PMC9356320

[12] Chua KP, Nguyen TD, Waljee JF, Nalliah RP, Brummett CM. Association between state opioid prescribing limits and duration of opioid prescriptions from dentists. JAMA Netw Open. 2023;6(1):e2250409. 36630136 10.1001/jamanetworkopen.2022.50409 PMC9857382

[13] McGinty EE, Bicket MC, Seewald NJ, Effects of state opioid prescribing laws on use of opioid and other pain treatments among commercially insured U.S. adults. Ann Intern Med. 2022;175(5):617–627. doi:10.7326/M21-4363 35286141 PMC9277518

[14] Osmundson SS, Wiese AD, Min JY, Delivery type, opioid prescribing, and the risk of persistent opioid use after delivery. Am J Obstet Gynecol. 2019;220(4):405–407. 30955527 10.1016/j.ajog.2018.10.026 PMC6546169

[15] Osmundson SS, Min JY, Wiese AD, Opioid prescribing after childbirth and risk for serious opioid-related events: a cohort study. Ann Intern Med. 2020;173(5):412–414. 32510992 10.7326/M19-3805 PMC8081555

[16] Wiese AD, Osmundson SS, Mitchel E Jr, Prescription opioid dose after vaginal delivery and the risk of serious opioid-related events: a retrospective cohort study. Womens Health Issues. 2021;31(4):376–383. 33865673 10.1016/j.whi.2021.03.002 PMC8260443

[17] Wiese AD, Osmundson SS, Mitchel E Jr, The risk of serious opioid-related events associated with common opioid prescribing regimens in the postpartum period after cesarean delivery. Am J Obstet Gynecol MFM. 2021;3 (6):100475. 34455101 10.1016/j.ajogmf.2021.100475 PMC8599660

[18] MacDonald SC, Cohen JM, Panchaud A, McElrath TF, Huybrechts KF, Hernández-Díaz S. Identifying pregnancies in insurance claims data: Methods and application to retinoid teratogenic surveillance. Pharmacoepidemiol Drug Saf. 2019;28(9):1211–1221. 31328328 10.1002/pds.4794 PMC6830505

[19] Davis CS, Lieberman AJ, Hernandez-Delgado H, Suba C. Laws limiting the prescribing or dispensing of opioids for acute pain in the United States: A national systematic legal review. Drug Alcohol Depend. 2019;194:166–172. 30445274 10.1016/j.drugalcdep.2018.09.022

[20] Dowell D, Ragan KR, Jones CM, Baldwin GT, Chou R. CDC clinical practice guideline for prescribing opioids for pain—United States, 2022. MMWR Recomm Rep. 2022;71(3):1–95. 10.15585/mmwr.rr7103a1 PMC9639433 36327391

[21] Reid DBC, Shah KN, Shapiro BH, Ruddell JH, Akelman E, Daniels AH. Mandatory prescription limits and opioid utilization following orthopaedic surgery. J Bone Joint Surg Am. 2019;101(10):e43. 31094987 10.2106/JBJS.18.00943

[22] Goodman-Bacon A Difference-in-differences with variation in treatment timing. J Econom. Published online June 12, 2021.

[23] Callaway B, Sant’Anna PHC. Difference-in-differences with multiple time periods. J Econom. Published online December 17, 2020.

[24] Zeldow B, Hatfield LA. Confounding and regression adjustment in difference-in-differences studies. Health Serv Res. 2021;56(5):932–941. 33978956 10.1111/1475-6773.13666 PMC8522571

[25] Du R, Ali MM, Sung YS, Maternal comorbidity index and severe maternal morbidity among Medicaid covered pregnant women in a US Southern rural state. J Matern Fetal Neonatal Med. 2023;36(1):2167073. 36683016 10.1080/14767058.2023.2167073

[26] American College of Obstetricians and Gynecologists. Pharmacologic stepwise multimodal approach for postpartum pain management: ACOG clinical consensus No. 1. Obstet Gynecol. 2021;138(3):507–517. 34412076 10.1097/AOG.0000000000004517

[27] Wing C, Freedman SM, Hollingsworth A. Stacked difference-in-differences. Accessed September 15, 2024. http://www.nber.org/papers/w32054

[28] Wooldridge JM. Two-way fixed effects, the two-way Mundlak regression, and difference-in-differences estimators. Accessed September 15, 2024.

[29] Allen LD, Pollini RA, Vaglienti R, Powell D. Opioid prescribing patterns after imposition of setting-specific limits on prescription duration. JAMA Health Forum. 2024;5(1):e234731. 38241057 10.1001/jamahealthforum.2023.4731 PMC10799257

[30] Jarlenski M, Bodnar LM, Kim JY, Donohue J, Krans EE, Bogen DL. Filled prescriptions for opioids after vaginal delivery. Obstet Gynecol. 2017;129(3):431–437. 28178050 10.1097/AOG.0000000000001868 PMC5321851

[31] Ali MM, West K, Nye E. Postpartum opioid prescription fills, opioid use disorder, and utilization of medication-assisted treatment among women with Medicaid and private health insurance coverage. Accessed September 15, 2024. https://aspe.hhs.gov/sites/default/files/migrated_legacy_files//197941/PostpartOUDIB.pdf

[32] Trost S, Beauregard J, Chandra G, Pregnancy-related deaths: data from maternal mortality review committees in 36 US states, 2017–2019. Accessed September 15, 2024. https://www.cdc.gov/maternal-mortality/php/data-research/mmrc-2017-2019.html

[33] Morgan JR, Leech AA. Commentary on Schmidt : Informed patient preference and prioritizing access to medications for opioid use disorder for pregnant individuals. Addiction. 2024;119(6):1123–1124. 38570825 10.1111/add.16495 PMC11781535

