# Supplementary material for: Changes in Postpartum Opioid Prescribing After Implementation of State Opioid Prescribing Limits
Source: JAMA Health Forum. Author manuscript; Available in PMC 2025 Feb 2. (PMC11787902; doi:10.1001/jamahealthforum.2024.4216)
Supplement: Supplement 1 — eTable 1. Diagnosis and procedure codes used for the identification of a delivery eTable 2. Identification of vaginal vs. cesarean births eTable 3. Status of state opioid prescribing limits used in analysis eAppendix. Identification of opioid analgesics eFigure 1. Logic followed for identification of opioid pain reliever NDCs in 2021 eTable 3. Generic opioid formulations included in the 2021 creation of the CDC Opioid Oral MME conversion table eTable 4. Categories and scores for maternal comorbidity index eTable 5. Diagnosis codes used for definition of mental health conditions and substance use disorder eFigure 2. Event study plot for Callaway-Sant’Anna estimator using the primary outcome, MMEs per delivery eTable 6. Unadjusted Callaway-Sant’Anna DID estimates compared to fully adjusted for the outcome, MMEs per delivery eTable 7. Average treatment effects using alternative DID estimators eReferences. [file NIHMS2047513-supplement-Supplement_1.pdf]

Supplemental Online Content

Fry CE, Jeffery AD, Horta M, et al. Changes in postpartum opioid prescribing after implementation of state opioid prescribing limits. *JAMA Health Forum*. 2024;5(11):e244216. doi:10.1001/jamahealthforum.2024.4216

eTable 1. Diagnosis and procedure codes used for the identification of a delivery
eTable 2. Identification of vaginal vs. cesarean births
eTable 3. Status of state opioid prescribing limits used in analysis
eAppendix. Identification of opioid analgesics
eFigure 1. Logic followed for identification of opioid pain reliever NDCs in 2021
eTable 3. Generic opioid formulations included in the 2021 creation of the CDC Opioid Oral MME conversion table
eTable 4. Categories and scores for maternal comorbidity index
eTable 5. Diagnosis codes used for definition of mental health conditions and substance use disorder
eFigure 2. Event study plot for Callaway-Sant'Anna estimator using the primary outcome, MMEs per delivery
eTable 6. Unadjusted Callaway-Sant'Anna DID estimates compared to fully adjusted for the outcome, MMEs per delivery
eTable 7. Average treatment effects using alternative DID estimators
eReferences.

This supplemental material has been provided by the authors to give readers additional information about their work.

© 2024 Fry CE et al. *JAMA Health Forum.*

We required one diagnosis or procedure code from eTable 1 in outpatient or inpatient claims to identify a delivery in our dataset. We then followed the algorithm provided in MacDonald et al. 2019 to differentiate between reasons why a pregnancy ended. From these reasons, we only included those that were the result of a delivery (i.e., not a spontaneous or elective abortion).

eTable 1. Diagnosis and procedure codes used for the identification of a delivery

Category	Codes
Diagnosis codes (ICD-9)	630, 6300, 632, 63300, 63310, 63320, 63380, 63390, 634, 6340, 63400, 63401, 63402, 6341, 63410, 63411, 63412, 6342, 63420, 63421, 63422, 6343, 63430, 63431, 63432, 6344, 63440, 63441, 63442, 6345, 63450, 63451, 63452, 6346, 63460, 63461, 63462, 6347, 63470, 63471, 63472, 6348, 63480, 63481, 63482, 6349, 63490, 63491, 63492, 635, 6350, 63500, 63501, 63502, 6351, 63510, 63511, 63512, 6352, 63520, 63521, 63522, 6353, 63530, 63531, 63532, 6354, 63540, 63541, 63542, 6355, 63550, 63551, 63552, 6356, 63560, 63561, 63562, 6357, 63570, 63571, 63572, 6358, 63580, 63581, 63582, 6359, 63590, 63591, 63592, 636, 6360, 63600, 63601, 63602, 6361, 63610, 63611, 63612, 6362, 63620, 63621, 63622, 6363, 63630, 63631, 63632, 6364, 63640, 63641, 63642, 6365, 63650, 63651, 63652, 6366, 63660, 63661, 63662, 6367, 63670, 63671, 63672, 6368, 63680, 63681, 63682, 6369, 63690, 63691, 63692, 637, 6370, 63700, 63701, 63702, 6371, 63710, 63711, 63712, 6372, 63720, 63721, 63722, 6373, 63730, 63731, 63732, 6374, 63740, 63741, 63742, 6375, 63750, 63751, 63752, 6376, 63760, 63761, 63762, 66231, 66950, 66951, 66960, 66961, 66970, 66971, 67912, 7614, 7630, 7632, 7633, 7634, 7636, 7651, 76510, 76511, 76512, 76513, 76514, 76515, 76516, 76517, 76518, 76519, 7660, 7661, 7662, 76621, 76622, 7680, 7796
Diagnosis codes (ICD-10)	O00, O000, O0000, O001, O00101, O00102, O00109, O0011, O002, O00201, O00202, O00209, O0021, O008, O0080, O009, O0090, O01, O010, O011, O019, O02, O020, O021, O028, O03, O030, O031, O032, O033, O0330, O0331, O0332, O0333, O0334, O0335, O0336, O0337, O0338, O0339, O034, O035, O036, O037, O038, O0380, O0381, O0382, O0383, O0384, O0385, O0386, O0387, O0388, O0389, O039, O045, O046, O047, O0480, O0481, O0482, O0483, O0484, O0485, O0486, O0487, O0488, O0489, O30001, O30002, O30003, O30101, O30102, O30103, O30201, O30202, O30203, O30801, O30802, O30803, O3091, O3092, O3093, O3101X0, O3102X0, O3103X0, O3111X0, O318X10, O318X20, O318X30, O357XX0, O364XX0, O4210, O42111, O42113, O4212, O480, O481, O601, O6010, O6010X2, O6010X3, O6010X4, O6010X5, O6012, O6012X0, O6012X4, O6012X5, O6012X9, O6013, O6013X0, O6013X4, O6013X5, O6014, O6014X0, O6014X4, O6014X5, O6014X9, O602, O6020, O6020X0, O6020X1, O6020X2, O6020X3, O6020X4, O6020X5, O6020X9, O6022, O6022X0, O6022X1, O6022X2, O6022X3, O6022X4, O6022X5, O6022X9, O6023, O6023X0, O6023X1, O6023X2, O6023X3, O6023X4, O6023X5, O6023X9, O61, O618, O62, O628, O63, O630, O631, O632, O639, O64, O640, O640XX1, O640XX2, O640XX3, O640XX4, O640XX5, O640XX9, O641, O641XX0, O641XX1, O641XX2, O641XX3, O641XX4, O641XX5, O641XX9, O642, O642XX0, O642XX1, O642XX2, O642XX3, O642XX4, O642XX5, O642XX9, O643, O643XX0, O643XX1, O643XX2, O643XX3, O643XX4, O643XX5, O643XX9, O644, O644XX0, O644XX1, O644XX2, O644XX3, O644XX4, O644XX5, O644XX9, O645, O645XX0, O645XX1, O645XX2, O645XX3, O645XX4, O645XX5, O645XX9, O648, O648XX0, O643XX3, O643XX4, O643XX5, O643XX9, O644, O644XX0, O644XX1, O644XX2, O644XX3, O644XX4, O644XX5, O644XX9, O645, O645XX0, O645XX1, O645XX2, O645XX3, O645XX4, O645XX5, O645XX9, O648, O648XX0,

© 2024 Fry CE et al. *JAMA Health Forum.*

	O648XX2, O648XX3, O648XX4, O648XX5, O648XX9, O649, O649XX1, O649XX2, O649XX3, O649XX4, O649XX5, O648XX1, O649XX9, O650, O651, O652, O653, O658, O662, O663, O664, O6641, O665, O666, O67, O69, O690, O690XX1, O690XX2, O690XX3, O690XX4, O690XX5, O690XX9, O691, O691XX1, O691XX2, O691XX3, O691XX4, O691XX5, O691XX9, O693, O693XX1, O693XX2, O693XX3, O693XX4, O693XX5, O693XX9, O694, O694XX1, O694XX2, O694XX3, O694XX4, O694XX5, O694XX9, O695, O695XX1, O695XX2, O695XX3, O695XX4, O695XX5, O695XX9, O698, O6981, O6981X1, O6981X2, O6981X3, O6981X4, O6981X5, O6981X9, O6982, O6982X1, O6982X2, O6982X3, O6982X4, O6982X5, O6982X9, O6989, O6989X1, O6989X2, O6989X3, O692, O692XX1, O692XX2, O692XX3, O692XX4, O692XX5, O692XX9, O6989X4, O6989X5, O6989X9, O699, O699XX1, O699XX2, O699XX3, O699XX4, O699XX5, O699XX9, O70, O702, O71, O710, O718, O7181, O72, O73, O74, O740, O744, O745, O746, O747, O755, O758, O7582, O77, O771, O778, O779, O80, O82, O862, O868, O87, O880, O8801, O881, O8811, O882, O8821, O883, O8831, O888, O8881, O89, O890, O8901, O893, O894, O895, O896, P014, P030, P032, P033, P034, P035, P0700, P0701, P0702, P0703, P0710, P0714, P0715, P0716, P0717, P0718, P0730, P080, P081, P0821, P0822, P95, V27, V270, V271, V272, V273, V274, V275, V276, V277, V279, V30, V300, V3000, V3001, V301, V302, V31, V310, V3100, V3101, V311, V312, V32, V320, V3200, V3201, V321, V322, V33, V330, V3300, V3301, V331, V332, V34, V340, V3400, V3401, V341, V342, V35, V350, V3500, V3501, V351, V352, V36, V360, V3600, V3601, V361, V362, V37, V370, V3700, V3701, V371, V372, V39, V390, V3900, V3901, V391, V392, Z332, Z370, Z371, Z372, Z373, Z374, Z3751, Z3752, Z3759, Z3761, Z3769, Z377, Z379, Z3800, Z3801, Z381, Z382, Z3830, Z3831, Z384, Z385, Z3861, Z3862, Z3864, Z3868, Z3869, Z387
Procedure codes	1960, 1961, 1963, 1965, 1966, 1967, 1968, 1969, 59120, 59121, 59130, 59135, 59136, 59140, 59150, 59400, 59409, 59410, 59414, 59510, 59514, 59515, 59525, 59610, 59612, 59614, 59620, 59622, 59812, 59820, 59821, 59830, 59840, 59841, 59850, 59851, 59852, 59855, 59856, 59857, 6662, 6901, 6951, 72, 720, 721, 722, 7221, 7229, 723, 7231, 7239, 724, 725, 7251, 7252, 7253, 7254, 726, 727, 7271, 7279, 728, 729, 730, 7301, 7309, 731, 732, 7322, 733, 734, 735, 7351, 7359, 736, 738, 739, 7391, 7392, 7393, 7394, 7399, 740, 741, 742, 743, 744, 749, 7491, 7499, 750, 754, 88016, 99464, S0190, S0199, S2260, S2262, S2265, S2266, S2267, 3E0P7VZ, 3E0P3VZ, 3E0DXGC, 3E063VJ, 3E060VJ, 3E053VJ, 3E050VJ, 3E043VJ, 3E040VJ, 3E033VJ, 3E030VJ, 10T28ZZ, 10T27ZZ, 10T24ZZ, 10T23ZZ, 10T20ZZ, 10S0XZZ, 10S07ZZ, 10J07ZZ, 10E0XZZ, 10D18Z9, 10D17Z9, 10D07Z8, 10D07Z7, 10D07Z6, 10D07Z5, 10D07Z4, 10D07Z3, 10D00Z2, 10D00Z1, 10D00Z0, 10A08ZZ, 10A07ZZ, 10A07ZX, 10A04ZZ, 10A03ZZ, 10A00ZZ, 10908ZC, 10908ZA, 10907ZC, 10907ZA, 10904ZC, 10903ZC, 10900ZC, 0W8NXZZ, 0UT64ZZ, 0UT60ZZ, 0UT54ZZ, 0UT50ZZ, 0UB68ZZ, 0UB67ZZ, 0UB64ZZ, 0UB63ZZ, 0UB60ZZ, 0UB58ZZ, 0UB57ZZ, 0UB54ZZ, 0UB53ZZ, 0UB50ZZ, 0U7C7ZZ, 0Q834ZZ, 0Q833ZZ, 0Q830ZZ, 0Q824ZZ, 0Q823ZZ, 0Q820ZZ

© 2024 Fry CE et al. *JAMA Health Forum.*

We used the diagnosis, procedure, and DRGs to identify vaginal vs. cesarean births. Where there was not a code to indicate which type of delivery, we assumed the delivery was vaginal given that cesarean births are reimbursed at a much higher rate than vaginal deliveries; thus, providers have financial incentive to code cesareans deliveries as such.

eTable 2. Identification of vaginal vs. cesarean births

	**Diagnosis Codes**	**Procedure Codes**	**DRGs**
Vaginal births	ICD-9: 650, V270, V271, V272, V273, V274, V275, V276, V277, V279, 66950, 66951, 7632<br><br>ICD-10: O80, Z370, Z371, Z372, Z373, Z374, Z3759, Z3769, Z377, Z379, O665, P032	ICD-9: 720, 721, 7221, 7229, 7231, 7239, 727, 7271, 7279, 728, 729<br><br>ICD-10: 10D07ZE, 0W8NXZZ, 10D07Z4, 10D07Z5, 10D07Z6, 10D07Z8<br><br>CPT: 59400, 59409, 59410, 59610, 59612, 59614	767, 774, 775, 796, 797, 798, 768, 805, 806, 807
Cesareans births		ICD-9: 740, 741, 742, 744, 749, 7499<br><br>ICD-10: 10D00Z0, 10D00Z1, 10D00Z2<br><br>CPT: 59510, 59514, 59515, 59620, 59622	765, 766, 783, 784, 785, 786, 787, 788

© 2024 Fry CE et al. *JAMA Health Forum.*

eTable 3. Status of state opioid prescribing limits used in analysis

	**Implementation date in analysis**	**States**
Opioid prescribing cap	Jan 2016	CT, MA, NY
	Jan 2017	AK, DE, IN, KY, LA, ME, MD, NH, NJ, OH, PA, RI, UT, VA, VT
	Jan 2018	AR, AZ, CO, FL, MI, MO, MS, NC, NV, OK, SC, TN, WV
	Jan 2019	MN, MT, TX, WA, WY
No opioid prescribing cap	--	AL, CA, DC, GA, IA, ID, KS, ND, NE*, NM, OR, SD, WI
Excluded	2012	IL**
Excluded	2017	HI***

SOURCES/NOTES: **Sources** Tormohlen et al. 2022; Davis et al. 2019; Stone et al. 2022; Schmid et al. 2022 **Notes** *Nebraska's opioid prescribing limit only applied to minors. Included as comparison state since sample was restricted to deliveries to enrollees ≥ 18 years. **Omitted: No pre-period data for Illinois, which implemented a prescribing cap in 2012. ***Omitted: Hawaii's prescribing cap is only for the co-prescribing of benzodiazepines and opioids, we omit if from all analyses.

© 2024 Fry CE et al. *JAMA Health Forum.*

eAppendix. Identification of opioid analgesics

National Drug Codes (NDCs) for opioid analgesics were identified using the CDC's Opioid Oral MME conversion table. However, this table has not been updated by the CDC since 2020. To identify NDCs for opioid analgesics in 2021, we searched the 2021 RedBook in MarketScan using generic drug names from the 2020 CDC list. Using the generic drug names and formulations, we excluded all NDCs for formulations that were not included in the CDC's Opioid Oral MME conversion table from 2020. eFigure 1 provides a flow chart of our logic to exclude NDCs. Included formulations and the number of associated NDCs are in eTable 4. MME conversion factors from the 2020 CDC file were extended to identified 2021 NDC numbers.

The file of opioid analgesics from the CDC included buprenorphine. We excluded rows that were not Belbuca, Butrans, or a generic extended-release buprenorphine patch; these medications are often used for the treatment of pain. Other forms of buprenorphine, however, are more commonly used for the treatment of opioid use disorder. This follows the guidance/recommendation of the CDC. Buprenex was also excluded because it's an injectable formulation not typically prescribed in an outpatient setting (i.e., not in the pharmacy claims). Buprenorphine patches and films were assigned MME conversion factors of 12.6 and 0.03 MMEs, respectively. All other NDCs were assigned the MME conversion factor from the CDC Opioid Oral MME Conversion table.

© 2024 Fry CE et al. *JAMA Health Forum.*

eFigure 1. Logic followed for identification of opioid pain reliever NDCs in 2021

All NDCs with a generic name including: codeine, fentanyl, hydrocodone, hydromorphone, methadone, morphine, oxycodone, oxymorphone, tapentadol, tramadol, buprenorphine, pentazocine, meperidine, propoxyphene, belladonna, butorphanol
N = 21,617

↓

Exclude cough/cold indications
N = 19,364

↓

Exclude all antidiarrheal, emetic indications
N = 19,301

↓

Exclude all sympathomimetic, vasodilating, anticholinergic, muscle relaxant indications
N = 19,273

↓

Exclude epidural, intrathecal, intramuscular, intravenous, & injectable formulations
N = 16,919

↓

Exclude powders & powders for solution
N = 16,448

↓

Exclude remaining NDCs with cough/cold medication ingredients*
N = 16,407

↓

Exclude food ingredients/creams
N = 16,398

↓

Exclude formulations for buprenorphine as MOUD
N = 15,790

© 2024 Fry CE et al. *JAMA Health Forum.*

eTable 3. Generic opioid formulations included in the 2021 creation of the CDC Opioid Oral MME conversion table

Generic drug name	# of NDCs	MME Conversion Factor
APC with codeine	7	0.15
acetaminophen/benzhydrocodone	6	1.0
acetaminophen/butalbital/codeine phosphate	1	0.15
acetaminophen/caffeine/dihydrocodeine bitartrate	62	0.15
acetaminophen/codeine phosphate	1480	0.15
acetaminophen/hydrocodone bitartrate	4976	1.0
acetaminophen/meperidine hydrochloride	1	0.1
acetaminophen/oxycodone hydrochloride	1181	1.5
acetaminophen/pentazocine hydrochloride	25	0.37
acetaminophen/propoxyphene hydrochloride	144	0.23
acetaminophen/propoxyphene napsylate	800	0.23
acetaminophen/tramadol hydrochloride	382	0.1
apap/butabarbital na/codeine phos	1	0.15
apap/butalbital/caff/codeine phos	55	0.15
apis mellifeca/belladonna/cantharis/mountain arnic	1	1.0
aspirin (buffered)/codeine phosphate	1	0.15
aspirin/butalbital/caffeine/codeine phosphate	110	0.15
aspirin/caffeine/dihydrocodeine bitartrate	14	0.15
aspirin/caffeine/propoxyphene hydrochloride	162	0.23
aspirin/carisoprodol/codeine phosphate	27	0.15
aspirin/codeine phosphate	270	0.15
aspirin/hydrocodone bitartrate	23	1.0
aspirin/oxycodone hcl/oxycodone terephthalate	20	1.5
aspirin/oxycodone hydrochloride	7	1.5
aspirin/pentazocine hydrochloride	1	0.37
belladonna	49	1.0
buprenorphine	67	0.03
butorphanol tartrate	14	7.0
celecoxib/tramadol hydrochloride	1	0.1
codeine phosphate	4	0.15
codeine sulfate	47	0.15
dihydrocodeine/apap/caffeine	1	0.15
fentanyl	330	7.2
fentanyl citrate	100	7.2
hydrocodone bitartrate	43	1.0
hydrocodone bitartrate/ibuprofen	263	1.0
hydromorphone hydrochloride	369	4.0
ibuprofen/oxycodone hydrochloride	6	1.5
meperidine hcl/promethazine hcl	21	0.1
meperidine hydrochloride	125	0.1
methadone hydrochloride	279	3.0
morphine sulfate	1110	1.0
morphine sulfate/naltrexone hydrochloride	18	1.0
oxycodone	6	1.5
oxycodone hydrochloride	1103	1.5
oxymorphone hydrochloride	206	3
propoxyphene hydrochloride	250	0.23

© 2024 Fry CE et al. *JAMA Health Forum.*

propoxyphene napsylate	29	0.23
tapentadol hydrochloride	68	0.4
tramadol hydrochloride	1108	0.1

© 2024 Fry CE et al. *JAMA Health Forum.*

Creation of modified maternal comorbidity index

Because our study spans the switch from ICD-9 to ICD-10, we created a modified maternal comorbidity index for 2015 to properly identify comorbidities across this switch and enrollees whose definition period was before the switch from ICD-9 to ICD-10 (October 2015) but gave birth after the switch. We follow the process laid out in Du et al (2023) to define the maternal comorbidity index using ICD-9 & ICD-10 codes.

The maternal comorbidity index used based on diagnosis codes that are put into 20 diagnostic categories with an additional 3 comorbidities reflecting the pregnant person's age. We included 21 of these categories and the diagnosis codes associated with each category used. We separated out mental health conditions and substance use disorders and accounted for those as standalone covariates because we felt that they represented meaningful confounders whose impact might be understated if included in the full comorbidity score.

We measured comorbidities using inpatient claims from the 90 days before delivery. The total possible score for the comorbidity index was 47.

eTable 4. Categories and scores for maternal comorbidity index

**Condition**	**Comorbidity score**
Severe preeclampsia or eclampsia	5
Congestive heart failure	5
Pre-existing hypertension	4
Congenital heart disease	4
Chronic ischemic heart disease	3
Sickle cell disease	3
Age ≥ 45	3
Valvular disease	2
HIV/AIDS	2
Mild or unspecified preeclampsia	2
Multiple gestation	2
Systemic lupus erythematosus	2
Placenta previa	2
Age 40-44	2
Pre-existing type II diabetes	1
Previous c-section	1
Gestational hypertension	1
Asthma	1
Age 35-39	1
Chronic renal disease	1
**TOTAL**	47

© 2024 Fry CE et al. *JAMA Health Forum.*

Definition of mental health conditions and substance use disorders for covariates
As described above, we removed several categories from the maternal comorbidity index to separately account for the prevalence of mental health conditions and substance use disorders. From the maternal comorbidity algorithm, we removed categories related to depression, psychoses, alcohol abuse, and drug abuse. We excluded diagnosis codes for nicotine use disorder, vaping, and those related to self-harm. We included diagnosis codes for ADHD and conduct disorders but excluded diagnosis codes related to developmental disorders (e.g., autism, dyslexia).

eTable 5. Diagnosis codes used for definition of mental health conditions and substance use disorder

Category	ICD-10 Codes	ICD-9 Codes
Substance use disorder	F1110, F11120, F11121, F11122, F11129, F1114, F11150, F11151, F11159, F11181, F11182, F11188, F1119, F1120, F11220, F11221, F11222, F11229, F1123, F1124, F11250, F11251, F11259, F11281, F11282, F11288, F1129, F1190, F11920, F11921, F11922, F11929, F1193, F1194, F11950, F11951, F11959, F11981, F11982, F11988, F1199, F1210, F12120, F12121, F12122, F12129, F12150, F12151, F12159, F12180, F12188, F1219, F1220, F12220, F12221, F12222, F12229, F12250, F12251, F12259, F12280, F12288, F1229, F12920, F12921, F12922, F12950, F12951, F12959, F12980, F12988, F1299, F1310, F13120, F13121, F13129, F1314, F13150, F13151, F13159, F13180, F13181, F13182, F13188, F1319, F1320, F13220, F13221, F13229, F13230, F13231, F13232, F13239, F1324, F13250, F13251, F13259, F1326, F1327, F13280, F13281, F13282, F13288, F1329, F1390, F13920, F13921, F13929, F13930, F13931, F13932, F13939, F1394, F13950, F13951, F13959, F1396, F1397, F13980, F13981, F13982, F13988, F1399, F1410, F14120, F14121, F14122, F14129, F1414, F14150, F14151, F14159, F14180, F14181, F14182, F14188, F1419, F1420, F14220, F14221, F14222, F14229, F1423, F1424, F14250, F14251, F14259, F14280, F14281, F14282, F14288, F1429, F1490, F14920, F14921, F14922, F14929, F1494, F14950, F14951, F14959, F14980, F14981, F14982, F14982, F14988, F1499, F1510, F15120, F15121, F15122, F15129, F1514, F15150, F15151, F15159, F15180, F15181, F15182, F15188, F1519, F1520, F15220, F15221, F15222, F15229, F1523, F1524, F15250, F15251, F15259, F15280, F15281, F15282, F15288, F1529, F1590, F15920, F15921, F15929, F1593, F1594, F15950, F15951, F15980, F15981, F15982, F15988, F1599, F1610, F16120, F16121, F16122, F16129, F1614, F16150, F16151, F16159, F16180, F16183, F16188, F1619, F1620, F16220, F16221, F16229, 1624, F16250, F16251, F16259, F16280, F16283, F16288, F1629, F1690, F16920, F16921, F16929,	292, 29211, 29212, 2922, 29281, 29282, 29283, 29284, 29285, 29289, 30400, 30401, 30402, 3041, 30411, 30412, 3042, 30421, 30422, 3043, 30431, 30432, 3044, 30441, 30442, 3045, 30451, 30452, 3046, 30461, 30462, 3047, 30471, 30472, 3048, 30481, 30482, 3049, 30491, 30492, 3052, 30521, 30522, 3053, 30531, 30532, 3054, 30541, 30542, 3055, 30551, 30552, 3056, 30561, 30562, 3057, 30571, 30572, 3058, 30581, 30582, 30590, 3059, 30591, 30592, 64830, 64831, 64832, 64833, 64834, 65550, 65551, 65553, 76072, 76073, 76075, 7795, 96500, 96501, 96502, 96509, E8541, E8500, E8501, E8502, E9359, E9351, V6542

© 2024 Fry CE et al. *JAMA Health Forum.*

	F1694, F16950, F16951, F16959, F16980, F16983, F16988, F1699, F18150, F18151, F18159, F1817, F18180, F18188, F1819, F1820 F18220, F18221, F18229, F1824, F18250, F18251, F18259, F1827, F18280, F18288, F1829, F1890, F18920, F18921, F18929, F182, F18280, F18288, F1829, F1890, F18920, F18921, F18929, F1894, F18950, F18951, F18959, F1897, F18980, F18988, F1899, F1910, F19120, F19122, F19129, F1914, F19150, F19151, F19159, F1916, F1917, F19180, F19181, F19182, F19188, F1919, F1920, F19220, F19221, F19222, F19229, F19230, F19231, F19232, F19239, F1924, F19250, F19251, F19259, F1926, F1927, F19280 F19281, F19282, F19288, F1929, F19230, F19231, F19232, F19239, F1924, F19250, F19251, F19259, F1926, F1927, F19280, F19281, F19282, F19288, F1929, F1990, F19920, F19921, F19922, F19929, F19930, F19931, F19932, F1994, F19950, F19951, F19959, F1996, F1997, F19980, F19981, F19982, F19988, F1999, F550, F551, F552, F553, F554, F558, OX355XX0, O355XX1, O355XX2, O355XX3, O355XX4, O355XX5, O355XX9, O99320, O99321, O99322, O99323, O99324, O99325, P0441, P0449, P961, P962, T400X1A, T400X2A, T400X3A, T400X4A, T400X5A, T400X5S, T401X1A, T401X2A, T401X3A, T401X4A, T402X1A, T402X2A, T402X3A, T402X4A, T403X1A, T403X2A, T403X3A, T403X4A, T403X5A, T403X5S, T404X1A, T404X2A, T404X3A, T404X4A, T40601A, T40602A, T40603A, T40604A, T40691A, T40692A, T40693A, T40694A, T407X1A, T408X1A, T40901A, T40991A, Z7141, Z7142 Z7151, Z7152, R4588, Z915, Z9151, Z9152, T5091X, U070	
Mental health condition	F200, F201, F202, F203, F205, F2081, F2089, F209, F250, F251, F258, F259, F4310, F4311, F4312, F060, F062, F2081, F21, F22, F23, F24, F28, F29, F322, F333, F4489, F340, F605, F605, F606, F607, F6081, F6089, F609, F6810, F6811, F6812, F6813, F69, F320, F321, F324, F325, F3289, F329, F330, F331, F332, F3340, F3341, F3342, F338, F339, F341, F313, F3131, F3132, F314, F315, F3160, F3161, F3162, F3163, F3164, F3175, F3176, F3177, F3178, F3181, F329, F43,21, F4323, F3010, F3011, F3012, F3013, F302, F303, F304, F308, F309, F310, F3110, F3111, F3112, F3113, F312, F315, F3160, F3161, F3162, F3163, F3164, F3170, F3171, F3172, F3173, F3174, F3175, F3176, F3177, F3178, F3181, F3189, F319, F338, F3481, F3489, F349, F39, F630, F631, F632, F633, F6381, F6389, F639, F900, F901, F902, F908, F909, F910, F911, F912, F913, F918, F919, F064, F4000, F4001, F4002, F4010, F4011, F40210, F40218, F40220, F40228, F40230, F40231, F40232, F40233, F40240, F40241, F40242, F40243, F40248, F40290, F40291, F40298, F408, F409, F410, F411, F413, F418, F419, F42, F422, F423, F424, F428, F429, F430, F4310,	312, 31201, 31202, 31203, 3121, 31211, 31212, 31213, 3122, 31221, 31222, 31223 3123, 31231, 31232, 31233, 31234, 31235, 31239, 3124, 31281, 31282, 31289, 3129, 314, 31401, 3141, 3142, 3148, 3149, 296, 29601, 29602, 29603, 29604, 29605, 29606, 2961, 29611, 29612, 29613, 29614 2964, 29641, 29642, 29643, 29644, 29645, 29646, 2965, 29651, 29652, 29653, 29654, 29655, 29656, 2966, 29661, 29662, 29663, 29664, 29665, 29666, 2967, 2968, 29681, 29682, 29689, 2969, 29699, 29384, 300, 30001, 30002, 30009, 3001, 3002X, 3003, 3005, 30089, 3009, 308, 3081, 3082, 3083,

© 2024 Fry CE et al. *JAMA Health Forum.*

	F4311, F4312, F449, F458, F488, F489, F938, F99, R452, R455, R456, R457, F630, F631, F632, F633, F6381, F6389, F639, F900, F901, F902, F908, F909, F910, F911, F912, F913, F918, F919, R4581, T1491, T1491XA, T1491XD, T1491XS, T360X2A, T360X2D, T360X2S, T361X2A, T361X2S, T361X2D, T363X2A, T363X2S, T363X2D, T364X2A, T364X2S, T364X2D, T365X2A, T365X2S, T365X2D, T366X2A, T366X2S, T366X2D, T367X2A, T367X2S, T367X2D, T368X2A, T368X2S, T368X2D, T369X2A, T369X2S, T369X2D, T370X2A, T370X2S, T370X2D, T371X2A, T371X2S, T371X2D, T372X2A, T372X2S, T372X2D, T373X2A, T373X2S, T373X2D, T374X2A, T374X2S, T374X2D, T375X2A, T375X2S, T375X2D, T378X2A, T378X2S, T378X2D, T379X2A, T379X2S, T379X2D, T380X2A, T380X2S, 380X2D, T381X2A, T381X2S, T381X2D, T382X2A, T384X2S, T384X2D, T385X2A, T385X2S, T385X2D, T386X2A, T386X2S, T386X2D, T387X2A, T387X2S, T387X2D, T38802A, T38802S, T38802D, T38812A, T38812S, T38812D, T38892A, T38892S, T38892D, T38902A, T38902D, T38902S, T38992A, T38992D, T38992S, T39012A, T39012D, T39012S, T39092A, T39092D, T39092S, T391X2A, T391X2D, T391X2S, T392X2A, T392X2D, T392X2S, T39312A, T39312D, T39312S, T394X2A, T394X2D, T394X2S, T398X2A, T398X2D, T398X2S, T3992XA, T3992XD, T3992XS, T400X2A, T400X2D, T400X2S, T401X2A, T401X2D, T401X2S, T402X2A, T402X2D, T402X2S, T403X2A, T403X2D, T403X2S, T404X2A, T404X2D, T404X2S, T405X2A, T405X2D, T405X2S, T40602A, T40602D, T40602S, T40692A, T40692D, T40692S, T407X2A, T407X2D, T407X2S, T408X2A, T408X2D, T408X2S, T40902A, T40902D, T40902S, T40992A, T40992D, T40992S, T410X2A, T410X2D, T410X2S, T411X2A, T411X2D, T411X2S, T41202A, T41202D, T41202S, T41292A, T41292D, T41292S, T413X2A, T413X2D, T413X2S, T4142XA, T4142XD, T4142XS, T415X2A, T415X2D, T415X2S, T420X2A, T420X2D, T420X2S, T421X2A, T421X2D, T421X2S, T422X2A, T422X2D, T422X2S, T423X2A, T423X2D, T423X2S, T424X2A, T424X2D, T424X2S, T425X2A, T425X2D, T425X2S, T426X2A, T426X2D, T426X2S, T4272XA, T4272XD, T4272XS, T428X2A, T428X2D, T428X2S, T43012A, T43012D, T43012S, T43022A, T43022D, T43022S, T431X2A, T431X2D, T431X2S, T43202A, T43202D, T43202S, T43212A, T43212D, T43212S, T43222A, T43222D, T43222S, T43292A, T43292D, T43292S, T433X2A, T433X2D, T433X2S, T434X2A, T434X2D, T434X2S, T43502A, T43502D, T43502S, T43592A, T43592D, T43592S, T43602A, T43602D, T43602S, T43612A, T43612D, T43612S, T43622A, T43622D, T43622S, T43632A, T43632D, T43632S, T43642A, T43642D, T43642S, T43692A, T43692D, T43692S, T438X2A, T438X2D, T438X2S, T4392XA, T4392XD, T4392XS, T440X2A, T440X2D, T440X2S, T441X2A, T441X2D, T441X2S, T442X2A,	3084, 3089, 30981, 313, 3131, 31321, 31322, 3133, 31382, 31383, 2962, 29621, 29622, 29633, 29634, 29635, 29636, 29651, 29652, 29653, 29654, 29655, 29656, 29660, 29661, 29662, 29663, 29664, 29665, 29666, 2980, 311, 31381, 30981, E9500, E9501, E9502, E9503, E9504, 39505, E9506, E9507, E9508, E9509, E9510, E9511, E9518, E9520, E9521, E9528, E9529, E9530, E9531, E9538, E9539, E954, E9550, E9551, E9552, E9553, E9554, E9555, E9556, E9557, E9559, E956, E9570, E9571, E9572, E9579, E9580, E9581, E9582, E9583, E9584, E9585, E9586, E9587 E9588, E9589, E959

© 2024 Fry CE et al. *JAMA Health Forum.*

	T442X2D, T442X2S, T443X2A, T443X2D, T443X2S, T444X2A, T444X2D, T444X2S, T445X2A, T445X2D, T445X2S, T446X2A, T446X2D, T446X2S, T447X2A, T447X2D, T447X2S, T448X2A, T448X2D, T448X2S, T44902A, T44902D, T44902S, T44992A, T44992D, T44992S, T450X2A, T450X2D, T450X2S, T451X2A, T451X2D, T451X2S, T452X2A, T452X2D, T452X2S, T453X2A, T453X2D, T453X2S, T454X2A, T454X2D, T454X2S, T45512A, T45512D, T45512S, T45522A, T45522D, T45522S, T45602A, T45602D, T45602S, T45612A, T45612D, T45612S, T45622A, T45622D, T45622S, T45692A, T45692D, T45692S, T457X2A, T457X2D, T457X2S, T458X2A, T458X2D, T458X2S, T4592XA, T4592XD, T4592XS, T460X2A, T460X2D, T460X2S, T461X2A, T461X2D, T461X2S, T462X2A, T462X2D, T462X2S, T463X2A, T463X2D, T463X2S, T464X2A, T464X2D, T464X2S, T465X2A, T465X2D, T465X2S, T466X2A, T466X2D, T466X2S, T467X2A, T467X2D, T467X2S, T468X2A, T468X2D, T468X2S, T46902A, T46902D, T46902S, T46992A, T46992D, T46992S, T470X2A, T470X2D, T470X2S, T471X2A, T471X2D, T471X2S, T472X2A, T472X2D, T472X2S, T473X2A, T473X2D, T473X2S, T474X2A, T474X2S, T48902S, T48992A, T48992D, T48992S, T490X2A, T490X2D, T490X2S, T491X2A, T491X2D, T491X2S, T492X2A, T492X2D, T492X2S, T493X2A, T493X2D, T493X2S, T494X2A, T494X2D, T494X2S, T495X2A, T495X2D, T495X2S, T496X2A, T496X2D, T496X2S, T497X2A, T497X2D, T497X2S, T498X2A, T498X2D, T498X2S, T4992XA, T4992XD, T4992XS, T500X2A, T500X2D, T500X2S, T501X2A, T501X2D, T501X2S, T502X2A, T502X2D, T502X2S, T503X2A, T503X2D, T503X2S, T504X2A, T504X2D, T504X2S, T505X2A, T505X2D, T505X2S, T506X2A, T506X2D, T506X2S, T507X2A, T507X2D, T507X2S, T508X2A, T508X2D, T508X2S, T50902A, T50902D, T50902S, T50992A, T50992D, T50992S, T50A12A, T50A12D, T50A12S, T50A22A, T50A22D, T50A22S, T50A92A, T50A92D, T50A92S, T50B12A, T50B12D, T50B12S, T50B92A, T50B92D, T50B92S, T50Z12A, T50Z12D, T50Z12S, T50Z92A. T50Z92D, T573X2A, T573X2D, T573X2S, T578X2A, T578X2D, T578X2S, T5792XA, T5792XD, T5792XS, T5802XA, T5802XD, T5802XS, T5812XA, T5812XD, T5812XS, T582X2A, T582X2D, T582X2S, T588X2A, T588X2D, T588X2S, T5892XA, T5892XD, T5892XS, T590X2A, T590X2D, T590X2S, T591X2A, T591X2D, T591X2S, T592X2A, T592X2D, T592X2S, T593X2A, T593X2D, T593X2S, T594X2A, T594X2D, T594X2S, T595X2A, T595X2D, T595X2S, T596X2A, T596X2D, T596X2S, T597X2A, T597X2D, T597X2S, T59812A, T59812D, T59812S, T59892A, T59892D, T59892S, T5992XA, T5992XD, T5992XS, T600X2A, T600X2D, T600X2S, T601X2A, T601X2D, T601X2S, T602X2A, T602X2D, T602X2S, T603X2A, T603X2D, T603X2S, T604X2A, T604X2D, T604X2S, T608X2A, T608X2D, T608X2S, T6092XA, T6092XD, T6092XS,	

© 2024 Fry CE et al. *JAMA Health Forum.*

	T6102XA, T6102XD, T6102XS, T6112XA, T6112XD, T6112XS, T61772A, T61772D, T61772S, T61782A, T61782D, T61782S, T618X2A, T618X2D, T618X2S, T6192XA, T6192XD, T6192XS, T620X2A, T620X2D, T620X2S, T621X2A, T621X2D, T621X2S, T622X2A, T622X2D, T622X2S, T628X2A, T628X2D, T628X2S, T6292XA, T6292XD, T6292XS, T63002A, T63002D, T63002S, T63012A, T63012D, T63012S, T63022A, T63022D, T63022S, T63032A, T63032D, T63032S, T63042A, T50Z92S, T510X2A, T510X2D, T510X2S, T511X2A, T511X2D, T511X2S, T512X2A, T512X2D, T512X2S, T513X2A, T513X2D, T513X2S, T518X2A, T518X2D, T518X2S, T5192XA, T5192XD, T5192XS, T520X2A, T520X2D, T520X2S, T521X2A, T521X2D, T521X2S, T522X2A, T522X2D, T522X2S, T523X2A, T523X2D, T523X2S, T524X2A, T524X2D, T524X2S, T528X2A, T528X2D, T528X2S, T5292XA, T5292XD, T5292XS, T530X2A, T530X2D, T530X2S, T531X2A, T531X2D, T531X2S, T532X2A, T532X2D, T532X2S, T533X2A, T533X2D, T533X2S, T534X2A, T534X2D, T534X2S, T535X2A, T535X2D, T535X2S, T536X2A, T536X2D, T536X2S, T537X2A, T537X2D, T537X2S, T5392XA, T5392XD, T5392XS, T540X2A, T540X2D, T540X2S, T541X2A, T541X2D, T541X2S, T542X2A, T542X2D, T542X2S, T543X2A, T543X2D, T543X2S, T5492XA, T5492XD, T5492XS, T550X2A, T550X2D, T550X2S, T551X2A, T551X2D, T551X2S, T560X2A, T560X2D, T560X2S, T561X2A, T561X2D, T561X2S, T562X2A, T562X2D, T562X2S, T563X2A, T563X2D, T563X2S, T564X2A, T564X2D, T564X2S, T565X2A, T565X2D, T565X2S, T566X2A, T566X2D, T566X2S, T567X2A, T567X2D, T567X2S, T56812A, T56812D, T56812S, T56892A, T56892D, T56892S, T5692XA, T5692XD, T5692XS, T570X2A, T570X2D, T570X2S, T571X2A, T571X2D, T571X2S, T572X2A, T572X2D, T572X2S, T63042D, T63042S, T63062A, T63062D, T63062S, T63072A, T63072D, T63072S, T63082A, T63082D, T63082S, T63092A, T63092D, T63092S, T63112A, T63112D, T63112S, T63122A, T63122D, T63122S, T63192A, T63192D, T63192S, T632X2A, T632X2D, T632X2S, T63302A, T63302D, T63302S, T63312A, T63312D, T63312S, T63322A, T63322D, T63322S, T63332A, T63332D, T63332S, T63392A, T63392D, T63392S, T63412A, T63412D, T63412S, T63422A, T63422D, T63422S, T63432A, T63432D, T63432S, T63442A, T63442D, T63442S, T63452A, T63452D, T63452S, T63462A, T63462D, T63462S, T63482A, T63482D, T63482S, T63512A, T63512D, T63512S, T63592A, T63592D, T63592S, T63612A, T63612D, T63612S, T63622A, T63622D, T63622S, T63632A, T63632D, T63632S, T63692A, T63692D, T63692S, T63712A, T63712D, T63712S, T63792A, T63792D, T63792S, T63812A, T63812D, T63812S, T63822A, T63822D, T63822S, T63832A, T63832D, T63832S, T63892A, T63892D, T63892S, T6392XA, T6392XD, T6392XS, T6402XA, T6402XD, T6402XS,	

© 2024 Fry CE et al. *JAMA Health Forum.*

	T6482XA, T6482XD, T6482XS, T650X2A, T650X2D, T650X2S, T651X2A, T651X2D, T651X2S, T65212A, T65212D, T65212S, T65222A, T65222D, T65222S, T65292A, T65292D, T65292S, T653X2A, T653X2D, T653X2S, T654X2A, T654X2D, T654X2S, T655X2A, T655X2D, T655X2S, T656X2A, T656X2D, T656X2S, T65812A, T65812D, T65812S, T65822A, T65822D, T65822S, T65832A, T65832D, T65832S, T65892A, T65892D, T65892S, T6592XA, T6592XD, T6592XS, T71112A, T71112D, T71112S, T71122A, T71122D, T71122S, T71132A, T71132D, T71132S, T71152A, T71152D, T71152S, T71162A, T71162D, T71162S, T71192A, T71192D, T71192S, T71222A, T71222D, T71222S, T71232A, T71232D, T71232S, X710XXA, X710XXD, X710XXS, X711XXA, X711XXD, X711XXS, X712XXA, X712XXD, X712XXS, X713XXA, X713XXD, X713XXS, X718XXA, X718XXD, X718XXS, X719XXA, X719XXD, X719XXS, X72XXXA, X72XXXD, X72XXXS, X730XXA, X730XXD, X730XXS, X731XXA, X731XXD, X731XXS, X732XXA, X732XXD, X732XXS, X738XXA, X738XXD, X738XXS, X739XXA, X739XXD, X739XXS, X7401XA, X7401XD, X7401XS, X7402XA, X7402XD, X7402XS, X7409XA, X7409XD, X7409XS, X748XXA, X748XXD, X748XXS, X749XXA, X749XXD, X749XXS, X75XXXA, X75XXXD, X75XXXS, X76XXXA, X76XXXD, X76XXXS, X770XXA, X770XXD, X770XXS, X771XXA, X771XXD, X771XXS, X772XXA, X772XXD, X772XXS, X773XXA, X773XXD, X773XXS, X778XXA, X778XXD, X778XXS, X779XXA, X779XXD, X779XXS, X780XXA, X780XXD, X780XXS, X781XXA, X781XXD, X781XXS, X782XXA, X782XXD, X782XXS, X788XXA, X788XXD, X788XXS, X789XXA, X789XXD, X789XXS, X79XXXA, X79XXXD, X79XXXS, X80XXXA, X80XXXD, X80XXXS, X810XXA, X810XXD, X810XXS, X811XXA, X811XXD, X811XXS, X818XXA, X818XXD, X818XXS, X828XXA, X828XXD, X828XXS, X830XXA, X830XXD, X830XXS, X831XXA, X831XXD, X831XXS, X832XXA, X832XXD, X832XXS, X838XXA, X838XXD, X838XXS	

© 2024 Fry CE et al. *JAMA Health Forum.*

Rationale for using non-yet-treated states as the comparison group:

With these estimators, researchers have the choice of comparison group – states that never implemented a prescription opioid limit or states that have not yet implemented a prescription opioid limit but do so in the future. Choosing states that never implement a limit gives a 'cleaner' comparison group – in other words, the comparison is being made to a group of states that represent a counterfactual world where the policy is not implemented, which is often the decision policymakers face. However, these states may be dissimilar to states that do implement a policy in ways that are not easily accounted for via covariate adjustment. Therefore, not-yet-treated states are potentially more like states that have implemented an opioid prescribing limit on observable and unobservable characteristics like the severity of the opioid crisis or political willingness to implement an opioid prescribing limit.

Additionally, the not-yet-treated comparison group is potentially more robust to anticipatory effects than a never-treated comparison group. In many instances, policies are passed several months before they are implemented, and prescribers may change their behavior before the implementation date in anticipation of the policy. Anticipatory effects in the treated group in the pre-period using a never-treated comparison group would bias results to the null. Conversely, anticipatory effects in the not-yet-treated comparison group would occur in the post-period and be used in the counterfactual estimation for the treated group, zeroing out the bias caused by anticipatory effects in the pre-period of the treated group. For these reasons, we prefer the not-yet-treated states as the comparison group.

© 2024 Fry CE et al. *JAMA Health Forum.*

eFigure 2. Event study plot for Callaway-Sant'Anna estimator using the primary outcome, MMEs per delivery

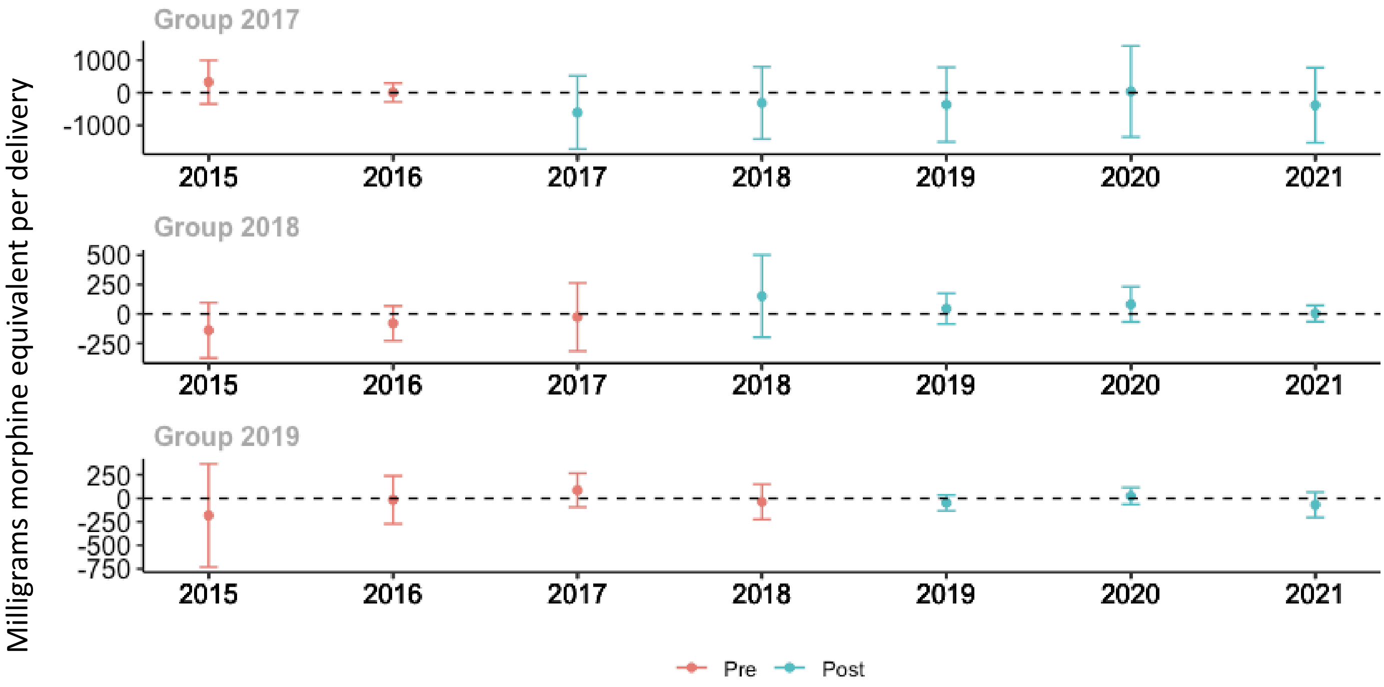

SOURCES/NOTES: **Sources** Authors' analyses of Meritav® MarketScan® claims data from 2013-2021. **Notes** Authors' analyses of Meritav® MarketScan® claims data from 2013-2021. **Notes** Values represent the covariate-adjusted DID estimates (average treatment effects on the treated; ATTs) from Callaway & Sant'Anna's staggered treatment timing estimator. Not-yet and never-treated states were included in the comparison group. Standard errors were clustered at the states level. Covariates included the average age at delivery, the proportion of enrollees with a mental health condition, the proportion of enrollees with a substance use disorder, and the average maternal comorbidity score. We identified opioid pain relievers by National Drug Codes. Calculations of milligrams of morphine equivalent (MMEs) for opioid pain relievers are calculated using the CDC's 2020 conversion table by active ingredient and day supply.

© 2024 Fry CE et al. *JAMA Health Forum.*

eTable 6. Unadjusted Callaway-Sant'Anna DID estimates compared to fully adjusted for the outcome, MMEs per delivery

	**Unadjusted**		**Adjusted**		**% difference**
All births	*Estimate*	*95% CI*	*Estimate*	*95% CI*	
**ATT**	**-122.69**	**-597.77, 352.40**	**-148.70**	**-657.97, 360.57**	**+21.2**
2017	-255.87	-1326.69, 814.94	-323.94	-1266.39, 618.52	+26.6
2018	-11.18	-107.56, 85.21	70.35	-60.15, 200.86	+729.25
2019	13.50	-60.45, 87.45	-31.92	-107.86, 44.02	-336.44

SOURCES/NOTES: **Sources** Authors' analyses of Meritav® MarketScan® claims data from 2013-2021. **Notes** Values represent the unadjusted* and covariate-adjusted** DID estimates from an extended two-way fixed effects regression. Regression models were adjusted for patient clinical characteristics (age, comorbidities, presence of a substance use disorder, or mental health condition). Standard errors were clustered by state. Definitions and diagnosis/procedure codes for births, vaginal births, and cesarean births are in the Appendix. We identified opioid pain relievers by National Drug Codes. Calculations of milligrams of morphine equivalent (MMEs) for opioid pain relievers are calculated using the CDC's 2020 conversion table by active ingredient and day supply

© 2024 Fry CE et al. *JAMA Health Forum.*

eTable 7. Average treatment effects using alternative DID estimators

	ETWFE		Stacked DID	
	Estimate	*95% CI*	*Estimate*	*95% CI*
Average Treatment Effect	-75.5	-331, 180	-165.79	-500.48, 158.90
Cohort effects				
2017	-216.0	-629.9, 378.0	-153.2	-427.60, 121.20
2018	-52.0	-145.6, 42.1	-208.5	-489.56, 72.56
2019	-32.3	-146, 81.6	-270.2	-650.44, 110.04

SOURCES/NOTES: **Sources** Authors' analyses of Meritav® MarketScan® claims data from 2014-2021. **Notes** Values represent the unadjusted DID estimates from an extended two-way fixed effects (ETWFE) and stacked DID regression. Standard errors were clustered by state. We identified opioid pain relievers by National Drug Codes. Calculations of milligrams of morphine equivalent (MMEs) for opioid pain relievers are calculated using the CDC's 2020 conversion table by active ingredient and day supply

© 2024 Fry CE et al. *JAMA Health Forum.*

eREFERENCES

Davis, Corey S., Amy Judd Lieberman, Hector Hernandez-Delgado, and Carli Suba. 2019. "Laws Limiting the Prescribing or Dispensing of Opioids for Acute Pain in the United States: A National Systematic Legal Review." *Drug and Alcohol Dependence* 194 (January):166–72. https://doi.org/10.1016/j.drugalcdep.2018.09.022.

MacDonald, Sarah C., Jacqueline M. Cohen, Alice Panchaud, Thomas F. McElrath, Krista F. Huybrechts, and Sonia Hernández-Díaz. 2019. "Identifying Pregnancies in Insurance Claims Data: Methods and Application to Retinoid Teratogenic Surveillance." *Pharmacoepidemiology and Drug Safety* 28 (9): 1211–21. https://doi.org/10.1002/pds.4794.

Schmid, Ian, Elizabeth A. Stuart, Alexander D. McCourt, Kayla N. Tormohlen, Elizabeth M. Stone, Corey S. Davis, Mark C. Bicket, and Emma E. McGinty. 2022. "Effects of State Opioid Prescribing Cap Laws on Opioid Prescribing after Surgery." *Health Services Research* 57 (5): 1154–64. https://doi.org/10.1111/1475-6773.14023.

Stone, Elizabeth M., Kayla N. Tormohlen, Alexander D. McCourt, Ian Schmid, Elizabeth A. Stuart, Corey S. Davis, Mark C. Bicket, and Emma E. McGinty. 2022. "Association Between State Opioid Prescribing Cap Laws and Receipt of Opioid Prescriptions Among Children and Adolescents." *JAMA Health Forum* 3 (8): e222461. https://doi.org/10.1001/jamahealthforum.2022.2461.

Tormohlen, Kayla N., Alex D. McCourt, Ian Schmid, Elizabeth M. Stone, Elizabeth A. Stuart, Corey Davis, Mark C. Bicket, and Emma E. McGinty. 2022. "State Prescribing Cap Laws' Association with Opioid Analgesic Prescribing and Opioid Overdose." *Drug and Alcohol Dependence* 240 (November):109626. https://doi.org/10.1016/j.drugalcdep.2022.109626.

© 2024 Fry CE et al. *JAMA Health Forum.*
