# Supplementary material for: Changes in Postpartum Opioid Prescribing After Implementation of State Opioid Prescribing Limits
Source: JAMA Health Forum. Author manuscript; Available in PMC 2025 Feb 2. (PMC11787902; doi:10.1001/jamahealthforum.2024.4216)
Supplement: Supplement 2 — Data sharing statement [file NIHMS2047513-supplement-Supplement_2.pdf]

Data Sharing Statement

Fry. Changes in Postpartum Opioid Prescribing After Implementation of State Opioid Prescribing Limits. *JAMA Health Forum*. Published November 27, 2024. doi:10.1001/jamahealthforum.2024.4216

Data
Data available: No
